# Atypical presentation of acute retinal necrosis mimicking Vogt-Koyanagi-Harada disease leading to misdiagnosis: a case report

**DOI:** 10.3389/fmed.2024.1468801

**Published:** 2024-11-22

**Authors:** Wei Zhu, Cuiyun Yu, Qianqian Guo, Qingran Kang, Xusheng Cao

**Affiliations:** ^1^Department of Ophthalmology, Zibo Central Hospital, Zibo, Shandong, China; ^2^Department of Outpatient, Zibo Central Hospital, Zibo, Shandong, China; ^3^Department of Laboratory Medicine, Shandong First Medical University, Jinan, Shandong Province, China; ^4^Department of Ophthalmology, Beijing Tongren Hospital, Beijing, China

**Keywords:** acute retinal necrosis (ARN), immunocompetent, Vogt-Koyanagi-Harada (VKH) disease, misdiagnosis, herpetic retinitis, antiviral therapy, case report

## Abstract

Acute retinal necrosis (ARN) is a serious, sight-threatening condition characterized by rapidly progressive necrotizing retinitis, most commonly caused by varicella-zoster virus and herpes simplex virus. We report an atypical case of ARN in a 57-year-old immunocompetent male, initially misdiagnosed as Vogt-Koyanagi-Harada (VKH) disease. This case highlights the challenges in the early differential diagnosis of infectious and non-infectious uveitis. Misdiagnosis can lead to a delay in initiating antiviral therapy, potentially accelerating disease progression and worsening visual outcomes.

## 1 Introduction

Acute retinal necrosis (ARN) represents a rapidly progressing necrotizing herpetic retinitis. It is primarily caused by varicella-zoster virus (VZV) and herpes simplex virus (HSV), and its clinical presentation is influenced by the degree of host immune response. ARN classically presents with prominent anterior chamber and vitreous inflammation, often accompanied by occlusive retinal vasculitis. Both immunocompromised and immunocompetent individuals can be affected. Diagnosing ARN can be difficult as many infectious and non-infectious conditions present similar symptoms and clinical features, such as CMV retinitis, toxoplasmic retinochoroiditis, syphilis, lupus vasculitis, Behçet’s disease, sarcoidosis, and bacterial or fungal chorioretinitis (1, 2). However, ARN mimicking the ophthalmologic findings of VKH is uncommon in clinical practice.

This report presents a case of ARN in an immunocompetent male, initially misdiagnosed as Vogt-Koyanagi-Harada (VKH) disease. This case underscores two crucial points: (1) the importance of a thorough differential diagnosis in suspected cases of uveitis; and (2) the potential for inappropriate corticosteroid treatment to exacerbate disease progression and mask classic clinical features.

## 2 Case description

**Figure 1:**
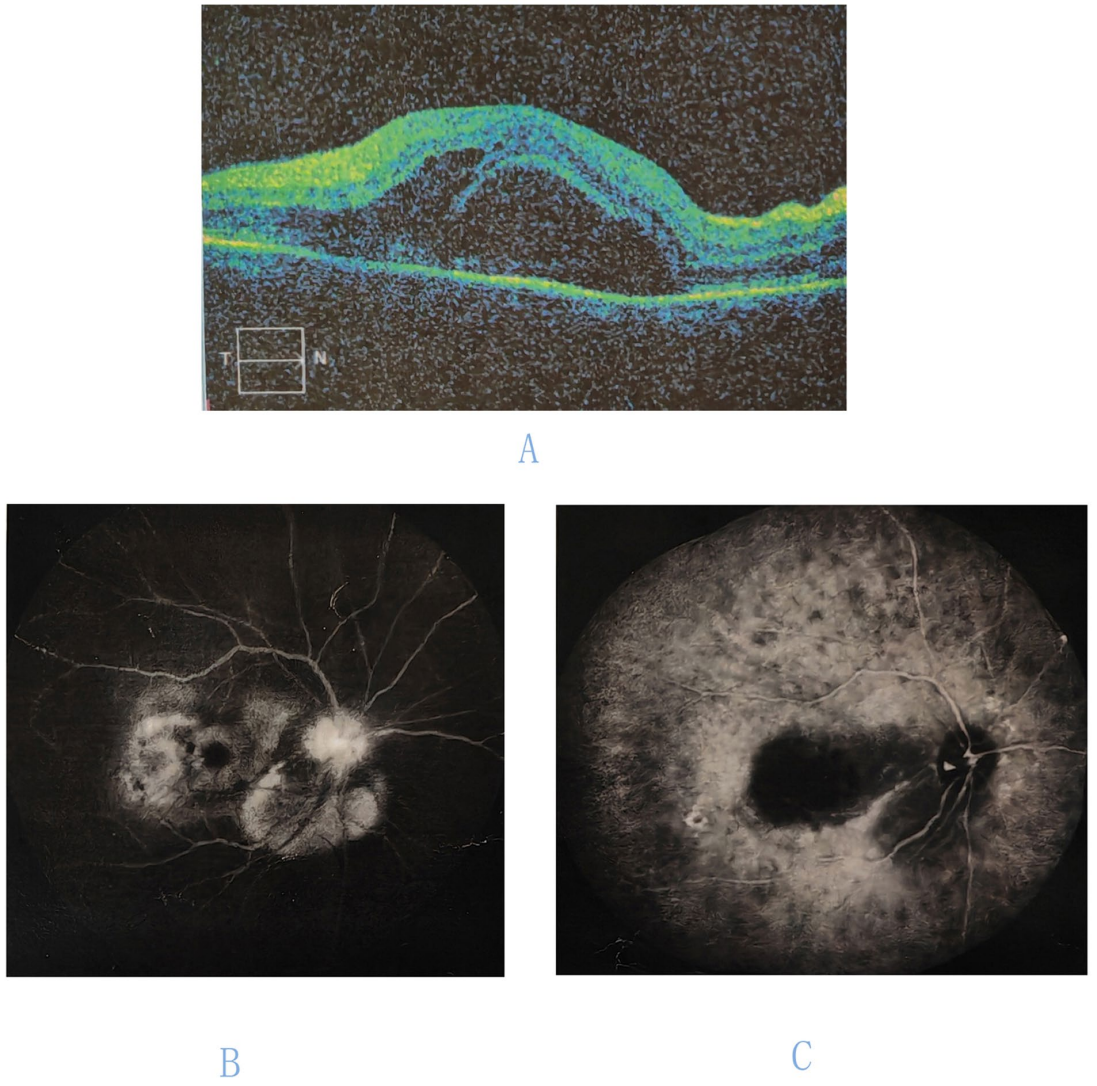
Ocular imaging of the right eye at initial presentation. **(A)** Optical coherence tomography (OCT) of the macula reveals subretinal fluid and intraretinal fluid. **(B)** Fluorescein angiography (FFA) demonstrates hyperfluorescence of the optic disk and multiple lake-like hyperfluorescent areas in the retina. **(C)** Indocyanine green angiography (ICGA) reveals hypofluorescence in the affected area.

**Figure 2:**
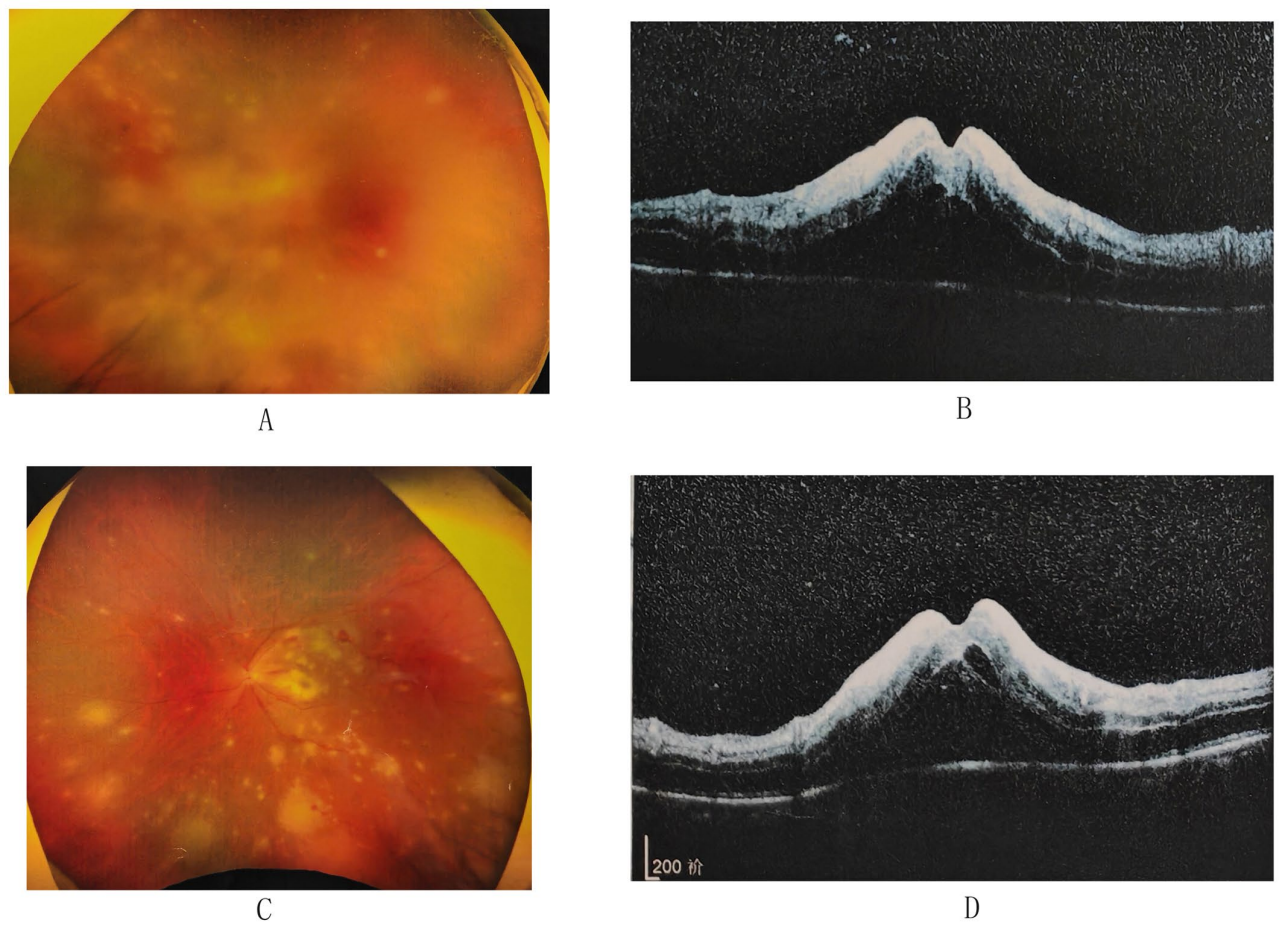
Fundus photography and optical coherence tomography (OCT) findings. **(A)** Fundus photograph of the right eye shows subtle yellowish-white, plaque-like lesions on the retina. **(B)** OCT of the right macula reveals subretinal fluid, intraretinal fluid, hyperreflective intraretinal layers, and retinal necrosis. **(C)** Fundus photograph of the left eye demonstrates bilateral optic disk edema with blurred margins. Multiple yellowish-white lesions are scattered throughout the posterior pole, periphery, and macula, involving the fovea. Some retinal vessels appear as white lines, indicating occlusion, and retinal hemorrhages are present. **(D)** OCT of the left macula reveals subretinal fluid, intraretinal fluid, hyperreflective intraretinal layers, and retinal necrosis.

A 57-year-old male presented with a 10-day history of progressively worsening bilateral blurred vision. Ten days prior to presentation at our institution, he presented to a local hospital. Fundus photography at that time revealed vitreous haze, blurred optic disc margins, scattered hemorrhages in the posterior pole, and yellowish-white lesions at the macula, and indistinct grayish-white lesions in the peripheral retina. Optical coherence tomography (OCT) showed subretinal fluid at the macula and intraretinal fluid. Late-phase fluorescein angiography (FFA) demonstrated hyperfluorescence of the optic disc and multiple lake-like hyperfluorescent areas in the retina. Indocyanine green angiography (ICGA) revealed hypofluorescence in the affected area and scattered hypofluorescent spots (Figure 1). Based on these findings, he was diagnosed with VKH disease and treated with oral prednisone 60 mg daily, topical prednisolone acetate eye drops three times daily in both eyes, and topical compound tropicamide eye drops twice daily in both eyes. Due to a lack of improvement, he presented to Beijing Tongren Hospital. He had no significant past medical history, denying any history of ocular disease, prior ocular surgery, or relevant family history. He denied recent travel, high-risk behaviors, tick bites, or intravenous drug use. Ocular examination findings are summarized in Table 1. Fundus photographs are shown in Figure 2.

**Table 1:** Ocular examination findings.

Examination type	Right eye	Left eye
Visual acuity	Counting fingers at face	Hand motion at face
Intraocular pressure	12 mmHg	10 mmHg
Anterior chamber	Reaction (++)	
Pupils	Pharmacologically dilated (approximately 8 mm)	
Vitreous	Mild vitritis (+)	
Fundus Examination(图2A)	Optic disc edema with blurred margins Multiple yellowish-white lesions scattered throughout the posterior pole, periphery, and macula, involving the fovea. Some retinal vessels appeared as white lines, indicating occlusion. Retinal hemorrhages present.	

Optical coherence tomography of the macula revealed subretinal fluid, intraretinal fluid, hyperreflective intraretinal layers, and retinal necrosis (Figure 2). Serological tests for antiphospholipid antibodies, anticardiolipin antibodies, antinuclear antibodies, anti-neutrophil cytoplasmic antibodies, as well as complete blood count, blood chemistry panel, tuberculosis screening, HIV, and syphilis IgG were all negative. Aqueous humor PCR analysis was strongly positive for VZV. The patient’s CD4+ T-cell count was 961 cells/μl, within the normal range.

**Figure 3:**
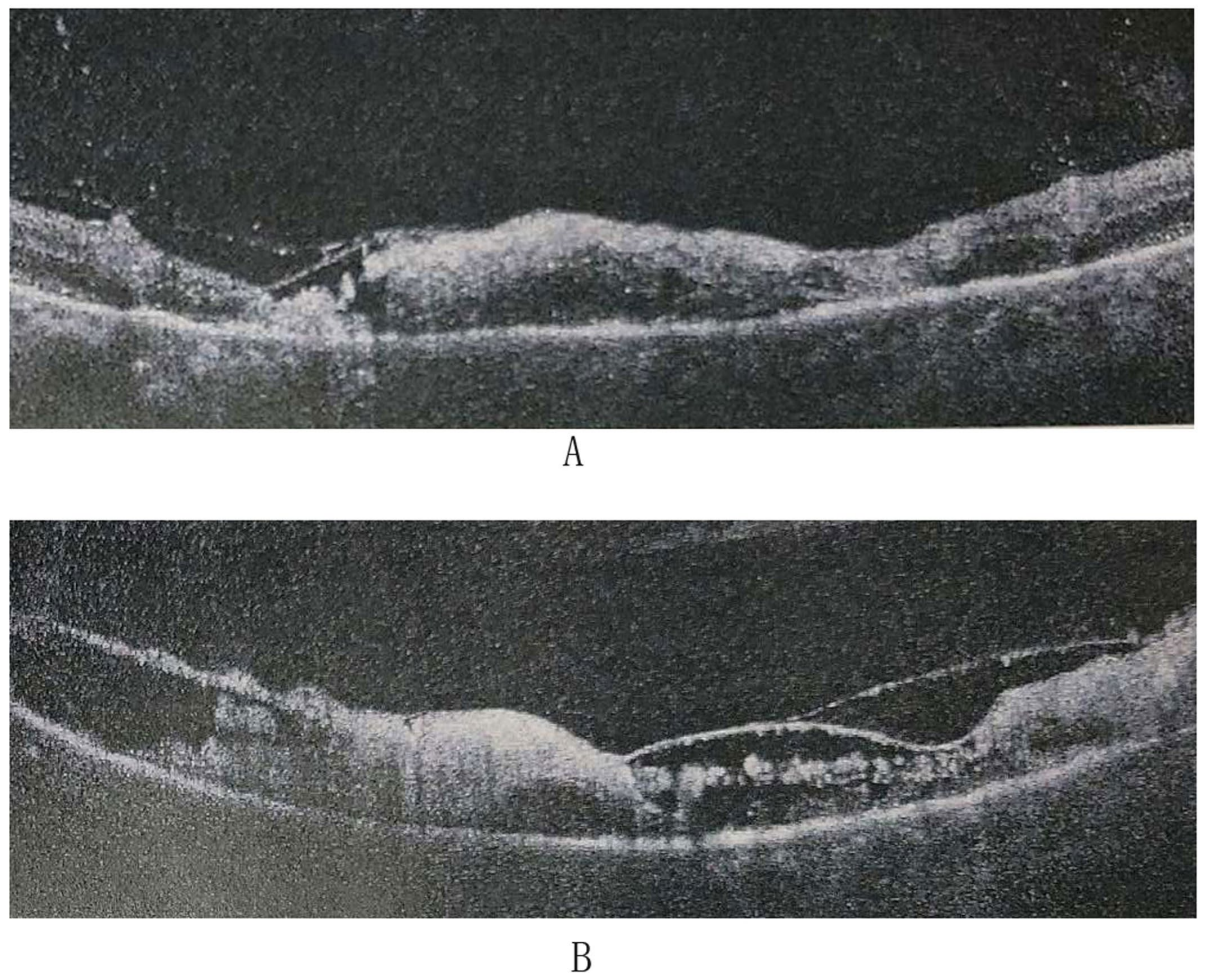
Optical coherence tomography (OCT) findings after treatment. **(A)** OCT of the right eye shows lysis of the inner and outer retinal structures in the macular region. **(B)** OCT of the left eye shows lysis of the inner and outer retinal structures in the macular region.

Based on the patient’s clinical presentation and laboratory findings, a diagnosis of Atypical ARN was made. Oral corticosteroid therapy (prednisone acetate tablets) was gradually tapered, and the patient was started on treatment with intravitreal ganciclovir 2 mg/0.1 mL every other day in both eyes and intravenous ganciclovir 0.25 g twice daily. At the 20-day follow-up, visual acuity was counting fingers at face in the right eye and no light perception in the left eye. OCT showed retinal retinolysis in both eyes (Figure 3).

## 3 Discussion

Acute retinal necrosis often presents with acute panuveitis. Clinical findings may include anterior uveitis, scleritis, vitritis, occlusive vasculitis, necrotizing retinitis, and optic disc edema (3). In the early stages of the disease, the patient’s OCT findings showed retinal detachment, subretinal membranous structures, and hyperreflective retinal spots. These findings closely resemble the characteristic OCT manifestations of VKH disease (4), contributing to the initial misdiagnosis. However, careful fundus examination revealed yellow-white lesions with relatively clear boundaries in the posterior pole and periphery. Detailed history taking revealed no symptoms of meningeal irritation, auditory abnormalities, or hair and skin changes. Additionally, the patient’s condition worsened after steroid use. These factors suggested that the patient did not have VKH disease. As observed in this case, choroidal involvement, characterized by elevation of the retinal pigment epithelium (RPE) layer on optical coherence tomography (OCT) and hypofluorescent lesions on indocyanine green angiography (ICGA), may be indicative of rapid disease progression in acute retinal necrosis (ARN) (5).

The misdiagnosis of VKH in this case led to the early administration of high-dose corticosteroids, thereby suppressing the body’s immune response to the virus. While this may have mitigated systemic and anterior segment inflammation, as well as retinal exudation, corticosteroid use alone, without concurrent effective antiviral therapy, can foster viral replication, delay optimal treatment, and accelerate disease progression (6). In this case, the early administration of corticosteroids may have contributed to the patient’s rapid progression of retinitis, resulting in severe and irreversible retinal damage, including retinal lysis and profound vision loss. The decision to initiate corticosteroid therapy in suspected cases of uveitis, particularly when the etiology is viral, presents a clinical dilemma. While corticosteroids can effectively reduce inflammation, their immunosuppressive effects could potentially exacerbate the underlying viral infection driving ARN. Delaying corticosteroid initiation may be beneficial in cases concerning infection (7). This underscores the importance of a comprehensive differential diagnosis and a careful assessment of the risks and benefits of corticosteroid therapy in such scenarios. Early intervention with antiviral therapy remains the cornerstone of ARN management (8). However, limitations in current antiviral therapy underscore the need for further investigation into novel therapeutic strategies to enhance visual prognosis. In this case, no significant visual improvement was observed during the follow-up period, but long-term follow-up is crucial to comprehensively assess the long-term visual outcome and monitor for any potential complications, such as retinal detachment.

In conclusion, this case highlights the importance of differentiating infectious from non-infectious uveitis in its early stages. The early presentation of choroidal inflammation suggests rapidly progressing ARN, and misdiagnosis can lead to treatment delays and potentially worsen outcomes.

## Data Availability

The raw data supporting the conclusions of this article will be made available by the authors, without undue reservation.
